# In vitro study on efficacy of PHELA, an African traditional drug against SARS-CoV-2

**DOI:** 10.1038/s41598-022-13599-y

**Published:** 2022-06-19

**Authors:** M. G. Matsabisa, K. Alexandre, Collins U. Ibeji, S. Tripathy, Ochuko L. Erukainure, K. Malatji, S. Chauke, B. Okole, H. P. Chabalala

**Affiliations:** 1 Department of Pharmacology, School of Medicine, Faculty of Health Sciences, University of the Free State, Bloemfontein, 9300 South Africa; 2 Synthetic Biology Centre, Next Generation Health Cluster, Council for Scientific and Industrial Research, Pretoria, Gauteng South Africa; 3 Department of Pure and Industrial Chemistry, Faculty of Physical Sciences, University of Nigeria, Nsukka, 410001 Nigeria; 4 Advanced Agriculture and Food Cluster, Council for Scientific and Industrial Research, Pretoria, South Africa; 5 Department of Science and Innovation, Indigenous Knowledge-based Technology Innovations, Brummeria, Pretoria, 0001 South Africa

**Keywords:** Receptor pharmacology, Metabolomics

## Abstract

In 2019, coronavirus has made the third apparition in the form of SARS-CoV-2, a novel strain of coronavirus that is extremely pathogenic and it uses the same receptor as SARS-CoV, the angiotensin-converting enzyme 2 (ACE2). However, more than 182 vaccine candidates have been announced; and 12 vaccines have been approved for use, although, even vaccinated individuals are still vulnerable to infection. In this study, we investigated PHELA, recognized as an herbal combination of four exotic African medicinal plants namely; *Clerodendrum glabrum E. Mey.* Lamiaceae*, Gladiolus dalenii* van Geel*, Rotheca myricoides* (Hochst.) Steane & Mabb, and *Senna occidentalis* (L.) Link; as a candidate therapy for COVID-19. In vitro testing found that PHELA inhibited > 90% of SARS-CoV-2 and SARS-CoV infection at concentration levels of 0.005 mg/ml to 0.03 mg/ml and close to 100% of MERS-CoV infection at 0.1 mg/ml to 0.6 mg/ml. The in vitro average IC_50_ of PHELA on SARS-COV-2, SARS-CoV and MERS-COV were ~ 0.01 mg/ml. Secondly in silico docking studies of compounds identified in PHELA showed very strong binding energy interactions with the SARS-COV-2 proteins. Compound 5 showed the highest affinity for SARS-COV-2 protein compared to other compounds with the binding energy of − 6.8 kcal mol^−1^. Our data showed that PHELA has potential and could be developed as a COVID-19 therapeutic.

## Introduction

SARS-CoV (Severe Acute Respiratory Syndrome Corona Virus) and MERS-CoV (Middle East Respiratory Syndrome Corona Virus) both posed major global health risks in 2002 and 2012, respectively. In 2019, coronavirus has made the third apparition in the form of SARS-CoV-2, a novel strain of coronavirus that is extremely pathogenic^1^. SARS-CoV-2 is an enveloped beta-coronavirus with non-segmented positive-sense RNA^2^. It infects people mostly through the respiratory system, and it uses the same receptor as SARS-CoV, the angiotensin-converting enzyme 2 (ACE2)^1,2^. The virus's surface spike (S) glycoprotein, membrane (M) protein, small envelope glycoprotein (E), and nucleocapsid (N) glycoproteins have all been identified as important therapeutic targets^1,3^. As a result, scientists are focusing their efforts on developing effective and reliable SARS-CoV-2 treatment alternatives^3^.

Vaccines and vaccine candidates are impressive not only in terms of quantity, but also in terms of diversity, as they include both standard (inactivated virion, live attenuated, and protein + adjuvant) and innovative (mRNA, DNA, and replicating and non-replicating viral vectors) platforms^4^. The fact that no protective correlation for any highly pathogenic coronavirus has been identified^5,6^, as well as the prospect that such correlates may differ amongst vaccines^7^ further complicates the situation. Nonetheless, it has been anticipated that both humoral and cell-mediated immunity can protect against coronaviruses^8,9^. Nonhuman primates have been verified to be protected from infection by antibody responses against the spike (S) protein^10,11^, and convalescent plasma with high anti-S antibody titers appear to have therapeutic potential in some patients^12^. Humoral responses in asymptomatic or mildly symptomatic persons can be mild and short-lived, disappearing within months of infection^13–15^ as seen during the SARS-CoV-1 outbreak in 2002–2003^16^. Not only for recovery from COVID-19, but also for long-term immunity, may an effective T cell response be necessary. T cells have been shown to exhibit long-lasting immunological responses to SARS-CoV-1^15,17^.

Plant products could be a good place to start looking for and developing anti-COVID-19 treatments. Plants have been utilized as medicines by people all over the world from ancient times, particularly in Asian countries such as India, China, and Japan, as well as in several African countries^18^. These herbs' broad availability and low cost have led to their popular use among indigenous people^19^. This gives hope that COVID-19 medications produced from natural products could be efficacious through a variety of ways^20–24^. Herbal medicines have also been used to treat viral diseases like SARS-CoV. According to the available data, herbal therapies may be useful in treating and reducing the risk of COVID-19. The Chinese National Health Commission has recognized the use of herbal medicine as a COVID-19 treatment option to Western medicine and has produced a number of herbal therapy guidelines^25^. Phytochemicals from medicinal plants such as alkaloids, flavonoids, polyphenols, tannins, lignins, coumarins, terpenoids, and stilbenes, are said to be useful in alleviating infections by binding to viral proteins and enzymatic activities, preventing viral penetration and replication in cells^19^. Many investigations have found that plant-derived bioactive compounds may have antiviral activities against the novel SARS-CoV-2^19,26^.

PHELA is recognized as an herbal combination of four exotic African medicinal plants namely, *Clerodendrum glabrum E. Mey.* Lamiaceae, *Gladiolus dalenii* van Geel*, Rotheca myricoides* (Hochst.) Steane & Mabb, and *Senna occidentalis* (L.) Link*.* These plants have traditionally been used as a remedy for wasting conditions, as an energy booster, and of recent, some traditional health practitioners claim that the preparation can help HIV/AIDS patients^27^. In a clinical study involving 500 HIV/AIDS patients, utilizing PHELA as an immune booster revealed an increase in appetite, 23% weight gain, and 80% reduction in viral load, and 200 percent increase in CD4 cell counts [The information acquired by an observational study of HIV positive and AIDS patients taking PHELA, a traditional Medicine, at Philisa Health Care Centre in Mabopane, North West province, South Africa from 2003 to 2005 by Matsabisa et al.,^28^]. PHELA is made by combining these distinct plants in a precise ratio of specific parts of these constituent plants^27–31^. A combination of these plants historically has been used to treat an ancient disease known as "Muyaga". Patients that contracted Muyaga were characterized by the following symptoms and signs: cough, shivering fever with headache, weight loss and loss of appetite, gastrointestinal disturbances, body rigidity and pains, and mouth ulcers. PHELA is presently under development by the University of Free State as an immune booster for HIV and its formulation has been confirmed to have immune-modulating properties^30^. PHELA reversed decreased white blood cell count, neutrophils, lymphocytes, and thymus weight in drug-suppressed immune system in rat models and had no harmful effects on the test animals in a subchronic toxicology study in vervet monkeys^29^. No toxicity was reported in phase I clinical trial in healthy human subjects (Medical Research Council, Indigenous Knowledge Systems Lead Programme report, 2009). In another study we conducted PHELA reversed both Dexamethasone and Cyclosporine induced immune suppression in study rats and was found to have no effect on a normal immune system.

Accumulating the previous reports on PHELA, here in this study we investigated PHELA as a repurposed product as a candidate therapy for COVID-19 in in vitro study and in silico docking study to evaluate its cytotoxicity, efficacy, and binding affinity to SARS-CoV-2 proteins. It is based on these data that we proposed PHELA to be repurposed for COVID-19.

## Materials and methods

### PHELA

The plants, *Clerodendrum glabrum E. Mey.* Lamiaceae (Voucher Specimen No.: BLFU MGM004), *Gladiolus dalenii* van Geel (Voucher Specimen No.: BLFU MGM001a and MGM001b), *Rotheca myricoides* (Hochst.) Steane & Mabb (Voucher Specimen No.: BLF/MGM0013), and *Senna occidentalis* (L.) Link (Voucher Specimen No.: BLFU/MGM0012) were collected with permission from the University Farm, Pannar Experimental Farm in Bainsvlei Bloemfontein, where the plants have been cultivated under controlled conditions. It was produced and supplied using strict good manufacturing standards by the Medical Research Council's Indigenous Knowledge Systems (IKS) Lead Programme (GMP). The relevant plant parts were dried and ground into a uniformly sized homogenous powder, which was subsequently sterilized using gamma radiation. PHELA was extracted in 70/30 ethanol/water with constant shaking over 24 h. PHELA is formulated in a specific ratio of the 4 plants part, stored for further uses and all the procedures were performed in accordance with the institutional and national guidelines and regulation.

### Compounds analysis and detection

Waters Ultra Performance Liquid Chromatography (UPLC) hyphenated with a Waters Synapt G2 QTOF instrument was used to analyse. Analysis was performed with Acquity UPLC BEH C18 1.7 µm (2.1 × 100 mm column), UPLC gradient mobile phase: A; water: 0.1% Formic Acid and B; Methanol + 0.1% HCO_2_H. The column operating flow rate was 0.300 mL/min.

### Cell lines and reagents

The 293-T ACE2.MF cells, enriched for the expression of human ACE2 receptor were a kind donation from Dr. Mike Farzan of Scripps Research Institute. Vero cells were obtained from the American Type Culture Collection. The cells were cultured in growth media (Dulbecco’s Modified Eagle’s Medium (DMEM) supplemented with 10% Fetal bovine serum and penicillin/streptomycin; and cells monolayer were disrupted at confluence by treatment with 0.25% trypsin in 1 mM EDTA. SARS-CoV-2 pseudovirus was made from a plasmid encoding SARS-CoV-2 envelope and a plasmid encoding the luciferase reporter gene (pNL4-3.luc.RE). The SARS-CoV-2 envelope genes used were from the Wuhan virus (GeneBank: MT039874.1), and first wave South Africa Virus (GeneBank: MT324062.1). The N501Y.V2 variant was made by introducing the mutations listed in Table 1 in the Wuhan virus using the Bioedit version 5.0.9.1 software and chemically synthesized by Genscript (GenScript, Piscataway, NJ, USA). SARS-CoV (GeneBank: DQ182595.1) and MERS-CoV (GeneBank: NC_019843.3) pseudoviruses were made the same as explained for SARS-CoV-2.

**Table 1:** List of mutations introduced in the Wuhan strain to make the N501Y.V2 variant.

Wuhan strain	Amino acid position on spike	N501Y.V2
L	18	F
D	80	A
D	215	G

		Δ242-244
K	417	N
E	484	K
N	501	Y
D	614	G
A	701	V

### Generation of SARV-CoV-2 and MERS-CoV pseudoviruses

SARS-CoV-2, SARS-CoV, and MERS-CoV were generated by co-transfection of the Envelope containing a plasmid, with a plasmid carrying the luciferase reporter gene as described by Wei et al.^32^. The co-transfection was done in 2 × 10^6^ 293-T ACE.MF cells/10 ml of growth media using the Fugene transfection reagent (Roche Applied Science, Indianapolis, IN). The TCID_50_ of the virus stock was quantified by infecting Vero cells with a serial fourfold dilution of the supernatant in quadruplicate in the presence of DEAE dextran (37.5 μg/ml) (Sigma-Aldrich, St. Louis, MO). The Bright Glo™ Reagent (Promega, Madison, WI) was used to measure infection after 72 h of tissue culture, according to the manufacturer's instructions. Luminescence was measured in the luminometer Tecan Infinite F500.

### Inhibition of different strains of coronaviruses infection of target cells

The 293-T ACE.MF cell line is used in studies investigating the virus-receptor interactions, and has a stable expression of the ACE2 receptor under the CMV promoter. These cells have been confirmed to express ACE2 at consistently high levels across multiple passages and are efficiently infected by SARS-CoV-2 pseudoviruses. We tested the ability of PHELA to neutralize SARS-CoV-2 infection of cells using the 293-T ACE. MF cells as targets. A 96 well plate was used in the inhibitory study, and the experiment was done in triplicates. Cell control, as well as virus control wells were included. A threefold serial dilution was performed by adding 50 µl of the extract to 100 µl of growth media. After the dilution series, 50 µl of SARS-CoV-2, SARS-CoV or MERS-CoV were added to all wells except the cell control wells. This was followed by incubation at 37 °C for 1 h to allow the sample to interact with the virus. Afterward 30,000 cells/100 µl/well of 293-T ACE.MF cells or Vero cells (for MERS-CoV) were added and centrifuged at 3500 rpm for 3 h and 30 min. After centrifugation, the plate was incubated at 37 °C, 5% CO_2_ and 95% humidity for 72 h. The incubation period was followed by the removal of 150 µl of growth media and the addition of 100 µl of Bright-Glo luciferase substrate to all the wells. Then the plate was incubated for 2 min at room temperature. Afterward, 150 µl was transferred to the corresponding wells of a 96-well black plate and luminescence was read using luminometer Tecan Infinite F500. IC_50_ values were calculated as the inhibitory concentration that causes 50% reduction of relative light unit (RLU) compared to the virus control (wells with no inhibitor) after the subtraction of the background (wells without both the virus and the inhibitor).

### Cytotoxicity of PHELA on 293T-ACE.MF cells

The cytotoxicity of PHELA on 293T-ACE.MF cells were, carried out by first seeding 30 000 cells/well/100 µl of the cells in a 96 well plate for 24 h at 37 °C, 5% CO_2_ and 95% humidity. After 24 h, a threefold serial dilution of PHELA extracts was performed and 100 µl was transferred to the plate containing cells, except in the cell control wells. The cells were then incubated for 72 h. Following the incubation, the spent media was removed and replaced with fresh 180 µl of growth media and 20 µl of 3-(4,5-Dimethylthiazol-2-yl)-2,5-diphenyltetrazolium bromide (MTT) reagent (5 mg/ml). This was followed by 3 h incubation at 37 °C, the removal of all media, and the addition of 150 µl of 0.1% triton X-Isopropanol. The plate was then incubated for 15 min at room temperature. Afterward, 100 µl of 4 mM hydrochloric acid was added and the plate was read at a wavelength of 620 nm using the Tecan Infinite F500 luminometer.

### Molecular docking

Molecular docking was carried out to determine the binding abilities of LC–MS identified compounds with the crystal structure of 2019-SARS-CoV-2 protein retrieved from the protein databank (http://www.rcsb.org/pdb) (PDB ID; 6M0J). AutoDock tools^33^ 1.5.4 was used to determine the suitable grid box size for the potential binding site. Gasteiger charges were computed using the AutoDock Tools graphical user interface implemented in MGL Tools^34^. The determined dimension was X = 24 Y = 24 Z = 24 with 1.00 Å as the grid spacing. The structures were optimized using Gaussian 09^35^, and the docking of ligand against each target was performed using AutoDock Vina^36^. The molecular docking protocol was validated by re-docking with Baricitinib (reference drug) with selected protein.

### Statistical analysis

In order to test the validity of obtained data, the statistical analysis was done using the graph pad prism7 software.

## Results

### PHELA compound analysis

UPLC MS Chromatograms were performed to analyze the compounds. The results have been shown in Table 2 (shows the selected active compounds).

**Table 2:** Chemical profile analysis of selected active compounds of PHELA (indicated #) and individual plants.

Sl. no	RT (min)	Acquired [M + H]^+^ m/z	Formula of possible structure	Theoretical [M + H]^+^ m/z	Calculated accurate mass (Da)	Mass error (ppm)	Possible structure
**Chemical profile of PHELA and *Senna occidentalis*, DCM extracts, ESI positive mode**							
1^#^	7.7	259.0968	C_15_H_14_O_4_	259.0970	258.0892	− 0.8	
1	7.7	259.0971	C_15_H_14_O_4_	259.0970	258.0892	0.4	
2^#^	8.3	269.0454	C_15_H_8_O_5_	269.0450	268.0371	1.5	
2	8.3	269.0459	C_15_H_8_O_5_	269.0450	268.0371	3.3	
**Chemical profile of PHELA and *Clerodendrum glabrum*, DCM extracts, ESI positive mode**							
3^#^	6.7	301.0705	C_16_H_12_O_6_	301.0712	300.0633	− 2.3	
3	6.7	301.0716	C_16_H_12_O_6_	301.0712	300.0633	− 2.3	
4^#^	8.3	331.1909	C_20_H_26_O_4_	331.1909	330.1831	0.0	
4	8.3	331.1906	C_20_H_26_O_4_	331.1909	330.1831	− 0.9	
5^#^	9.42	347.1862	C_20_H_26_O_5_	347.1858	346.1780	1.2	
5	9.42	347.1851	C_20_H_26_O_5_	347.1858	346.1780	− 2.0	
**Chemical profile of PHELA and *Rotheca myriciodes*, DCM extracts, ESI positive mode**							
6^#^	9.8	349.2017	C_20_H_28_O_5_	349.2015	348.1936	0.6	
6	9.8	349.2015	C_20_H_28_O_5_	349.2015	348.1936	0.0	
**Chemical profile of PHELA and *Gladiolus dalenii*, DCM extracts, ESI negative mode**							
7^#^	10.84	331.1916	C_20_H_28_O_4_	331.1909	332.1987	2.1	
7	10.85	331.1906	C_20_H_28_O_4_	331.1909	332.1987	− 0.9	
**Chemical profile of PHELA and *Rotheca myriciodes*, DCM extracts, ESI negative mode**							
8^#^	12.8	317.2121	C_20_H_30_O_3_	317.2117	318.2194	− 0.9	
8	12.8	317.2117	C_20_H_30_O_3_	317.2117	318.2194	0.0	
**Chemical profile of PHELA and *Clerodendrum glabrum*, MeOH extracts, ESI negative mode**							
9^#^	0.6	341.1089	C_12_H_22_O_11_	341.1084	342.1162	1.5	
9	0.6	341.1081	C_12_H_22_O_11_	341.1084	342.1162	− 0.9	

### Inhibitory effect of PHELA on different strains of SARS-CoV-2

**Figure 1:**
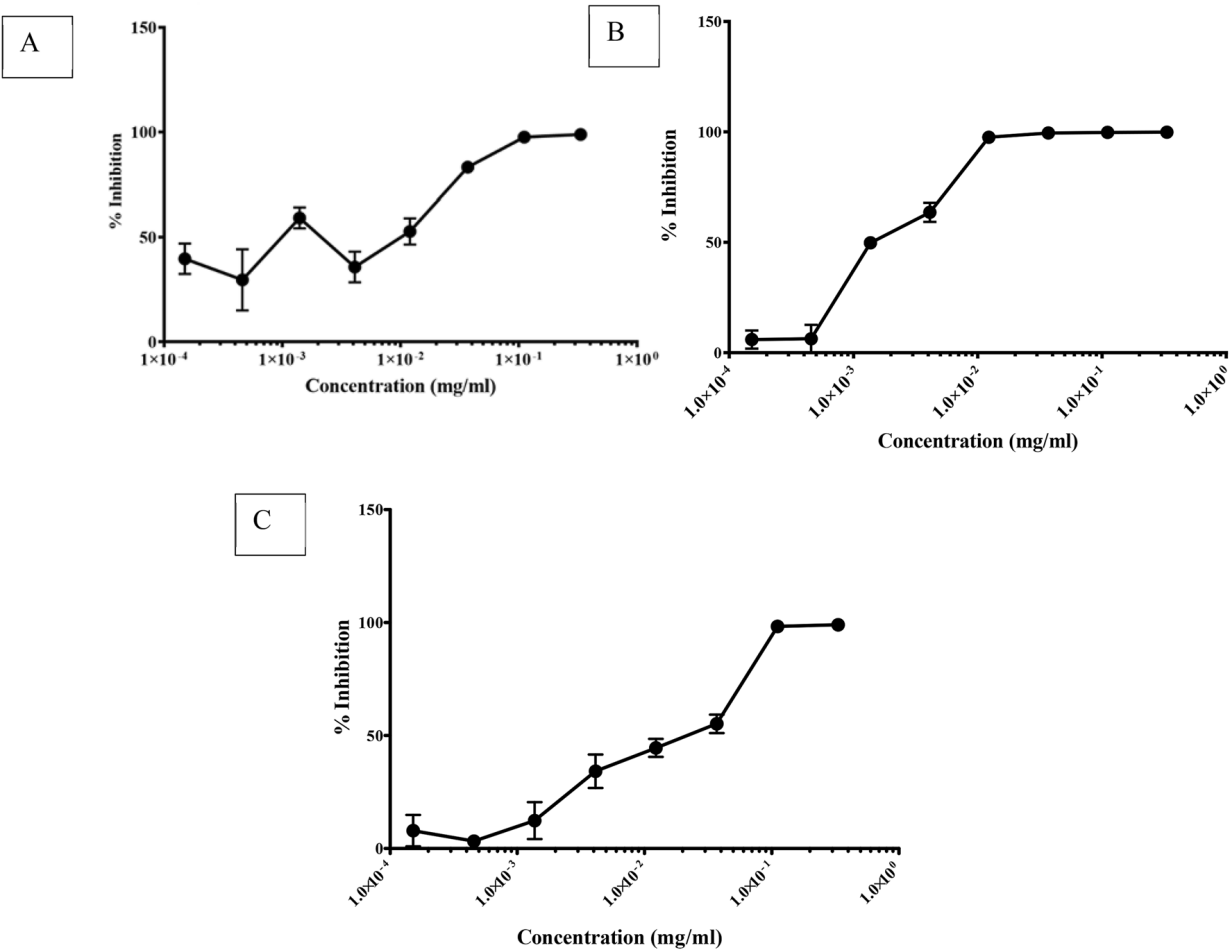
PHELA extract inhibition of SARS-CoV-2. (**A**) Wuhan strain, (**B**) a South African strain isolated during the first wave of the pandemic, and (**C**) the N501Y.V2 variant were incubated with the extract before infection of 293-T cells ACE.MF. After 72 h incubation the inhibition of infection was determined by measuring luminescence. Data shown represent the results of three independent experiments, and bars represent the means ± standard deviation.

**Figure 2:**
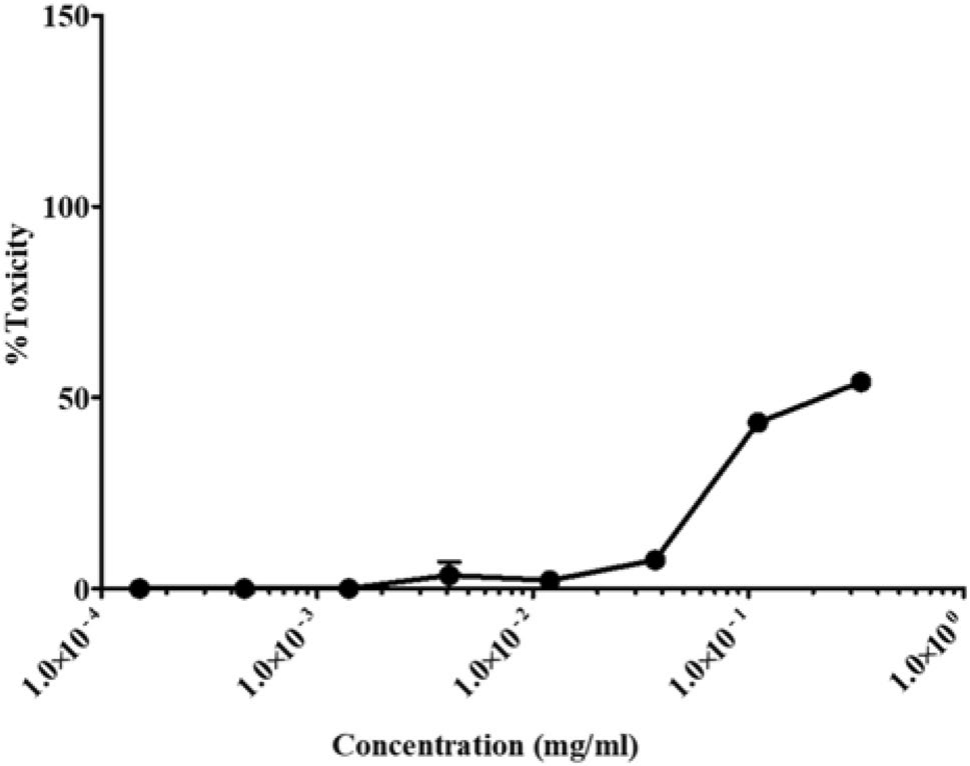
Evaluation of the PHELA extract cytotoxicity in 293-T ACE.MF cells. The extract was incubated with 293T-ACE.MF cells for 72 h followed by the determination of cytotoxicity using the MTT assay. Data shown are means ± standard deviation of three independent experiments.

We used the SARS-CoV-2 Wuhan strain that initiated the pandemic in 2019^37,38^, a strain isolated in South Africa during the first wave, as well as the 501Y.V2 variant strain. As shown in Fig. 1, the extract inhibited all SARS-CoV-2 entry into 293-T ACE.MF cells. Furthermore, at the highest concentration of PHELA tested we observed a 100% inhibition of all strains tested. Comparing the sensitivity of each strain to the inhibitor, the South Africa strain was the most sensitive with an IC_50_ value of 0.00134 mg/ml, while the Wuhan and the 501Y.V2 strains had IC_50_ values of 0.0100 mg/ml and 0.0241 mg/ml, respectively (Table 3). PHELA also showed a low level of toxicity to the cells, as determined by MTT assay, with a TC_50_ value of 0.476 mg/ml (also see Fig. 2); while at concentrations used in the study, the extract was not toxic. Nevertheless, these data indicate that PHELA is active against SARS-CoV-2 with IC_50_ values in the low microgram range.

**Table 3:** IC_50_ values of PHELA inhibition of different strains of SARS-CoV-2.

Envelope	50% inhibition (IC_50_) (mg/ml)
Wuhan strain	0.0100
South African strain	0.00134
501Y.V2 strain	0.0241

### Inhibitory effect of PHELA on coronavirus family

**Figure 3:**
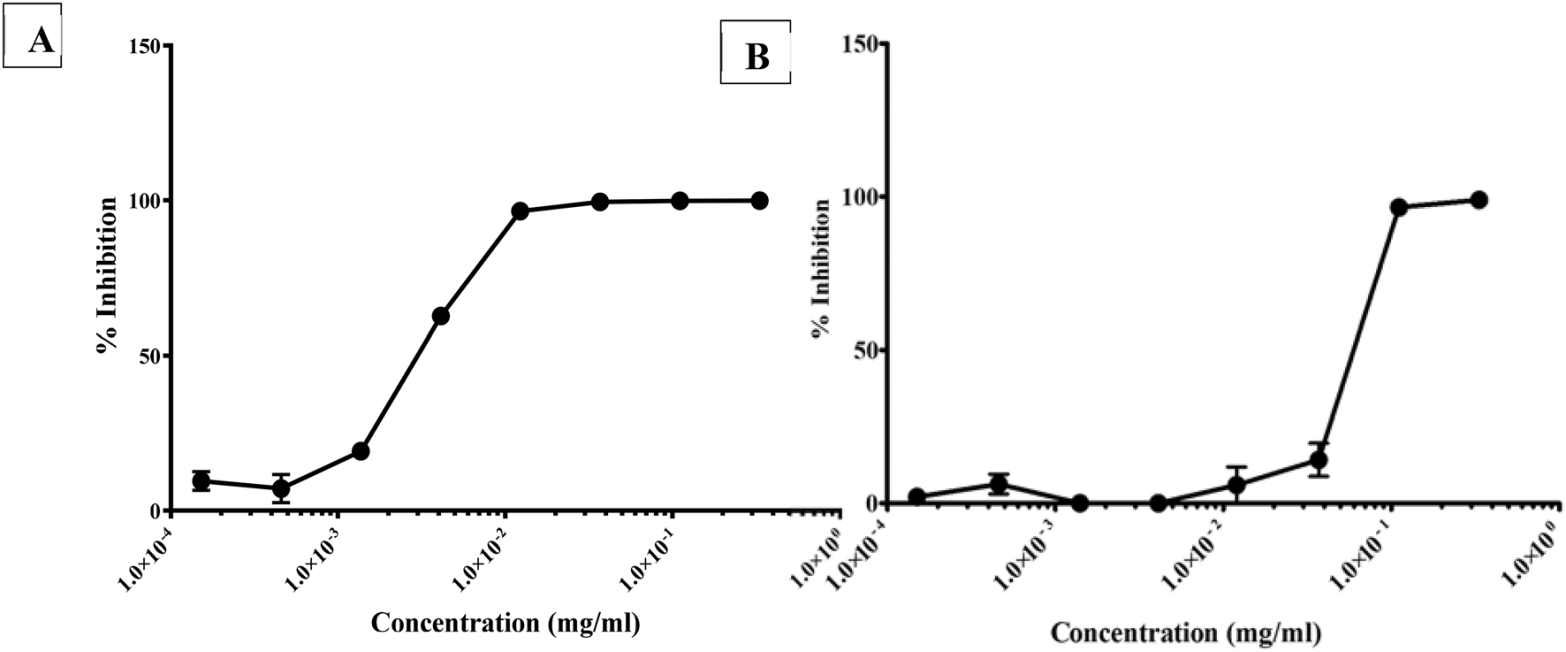
PHELA extract inhibition of SARS-CoV related coronaviruses. (**A**) SARS-CoV, and (**B**) MERS-CoV were incubated with the extract before infection of 293-T cells ACE.MF or Vero cells, respectively. After 72 h incubation the inhibition of infection was determined by measuring luminescence. Data shown represent the results of three independent experiments, and bars represent the means ± standard deviation.

In fact, SARS-CoV has an 80% genetic similarity to SARS-CoV-2, while MERS-CoV is around 50% genetically similar to this virus. We tested these viruses for susceptibility to PHELA using 293-T ACE.MF cells for SARS-CoV and Vero cells for MERS-CoV. Like what we observed for SARS-CoV-2, SARS-CoV and MERS-CoV, these viruses were sensitive to PHELA with 100% inhibition observed at the highest concentration of the extract used (Fig. 3). In addition, the inhibition of SARS-CoV was achieved with an IC_50_ value of 0.00348 mg/ml, while MERS-CoV was inhibited with an IC_50_ of 0.05900 mg/ml (Table 4), implying that the former was 16 times more sensitive to PHELA than the later. The above data suggest that PHELA has potential for use against other members of the coronavirus family that are also known to cause the severe acute respiratory syndrome.

**Table 4:** IC_50_ values of PHELA inhibition of SARS-CoV and MERS-CoV.

Envelope	50% inhibition (IC_50_) (mg/ml)
SARS-CoV-1	0.00348
MERS-CoV	0.0590

### Molecular docking

**Figure 4:**
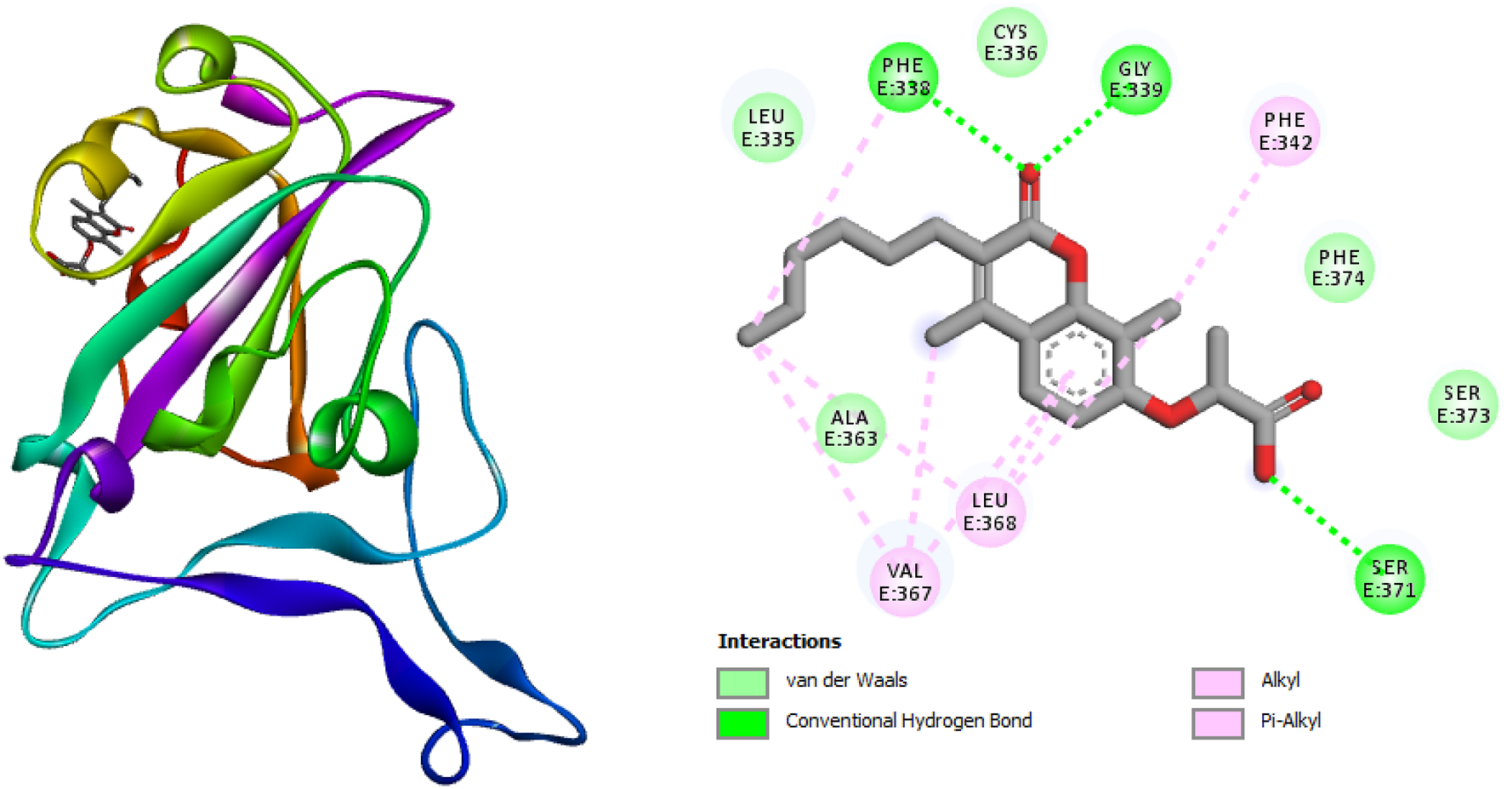
Three dimension (3D) structure of (**A**) Compound 5 in complex with SARS-CoV-2 protein (highest binding energy) (**B**) 2-D representations of docked complex of Compound 5 in complex SARS-CoV-2 protein displaying the interactions with amino acid residues.

**Figure 5:**
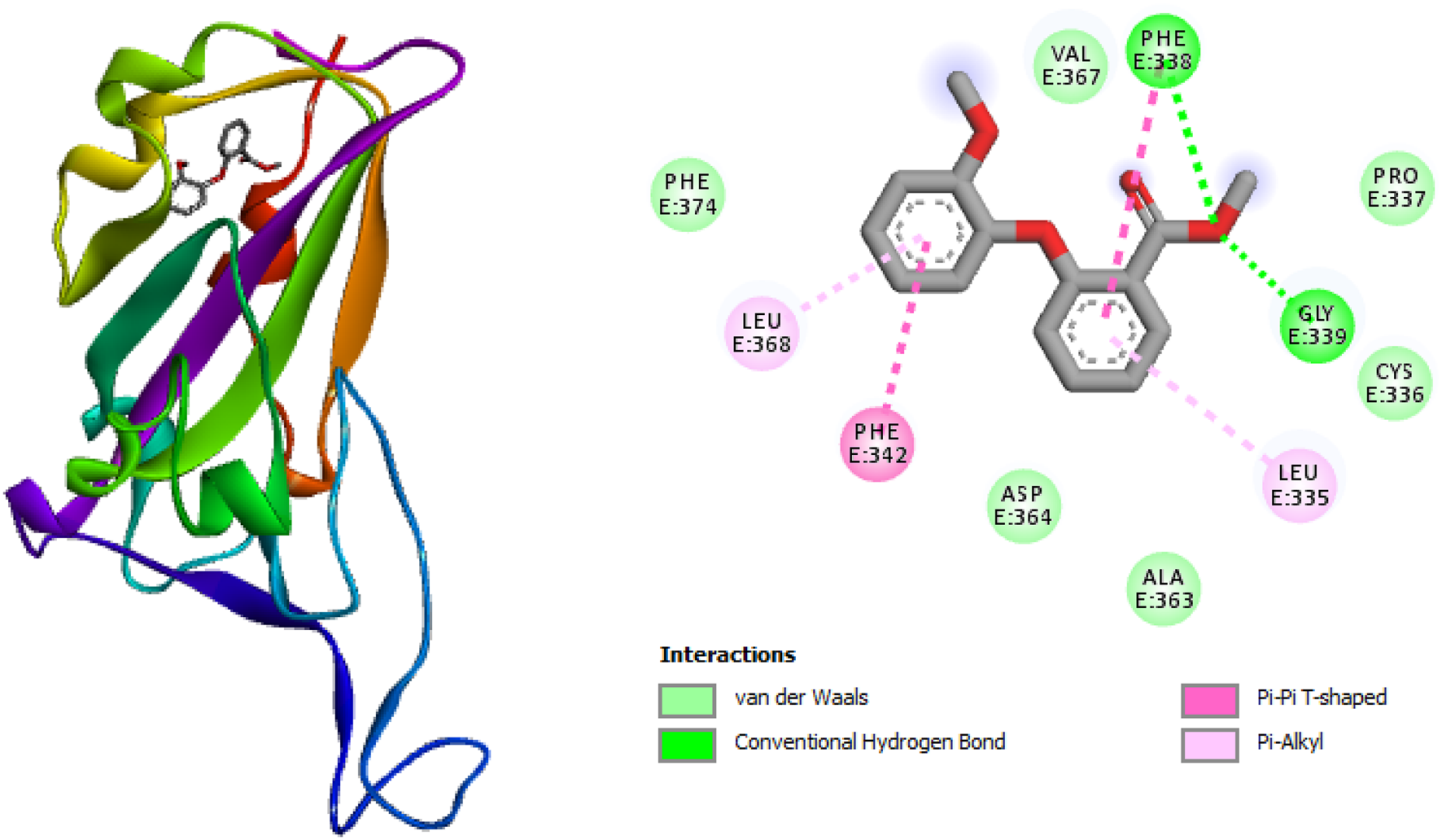
Three dimension (3D) structure of (**A**) Compound 1 in complex with SARS-CoV-2 protein (highest binding energy) (**B**) 2-D representations of a docked complex of Compound 1 in complex SARS-CoV-2 protein displaying the interactions with amino acid residues.

**Figure 6:**
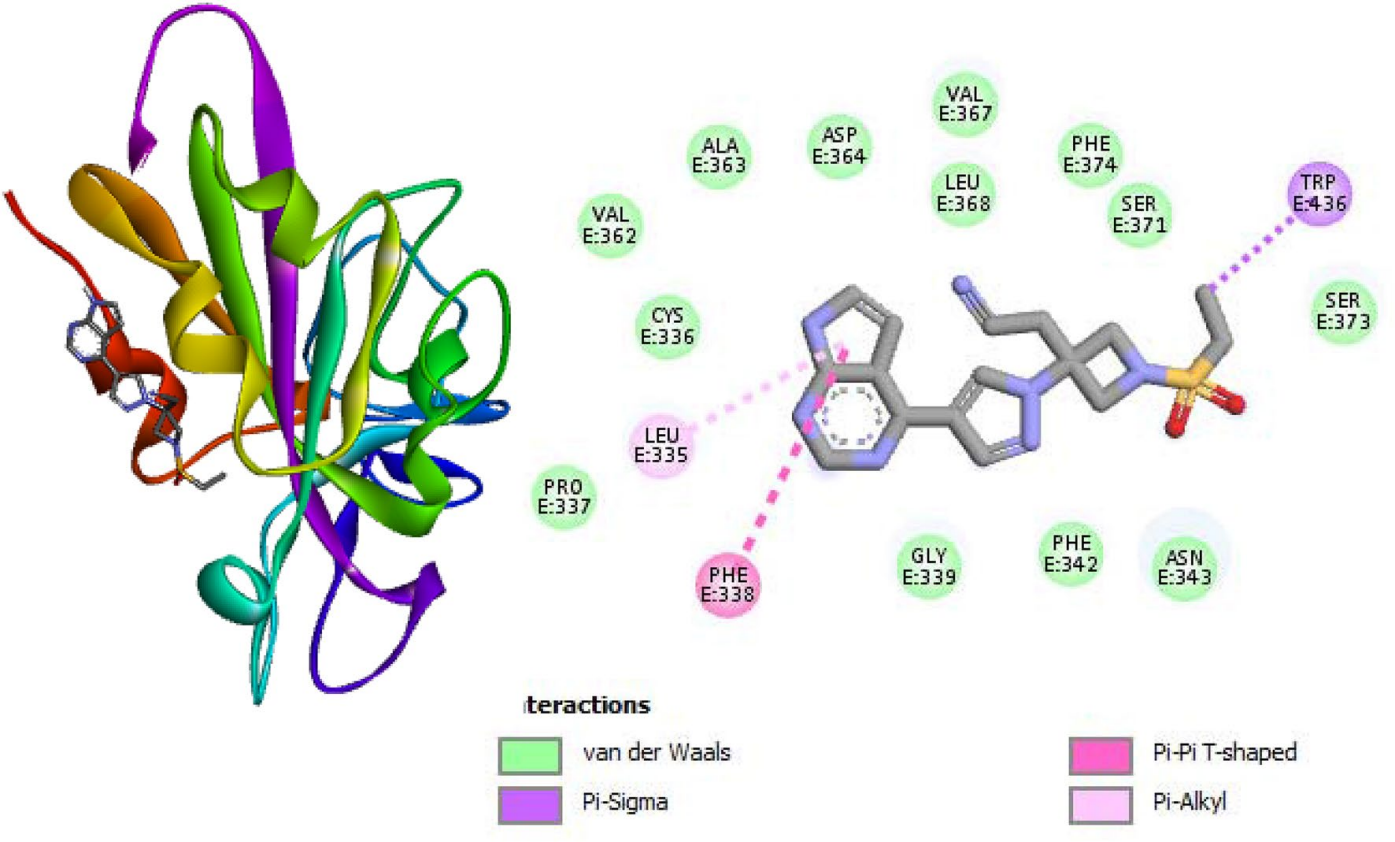
3D structure of (**A**) Baricitinib in complex SARS-CoV-2 protein (**B**) 2-D representations of a docked complex of Baricitinib in complex SARS-CoV-2 protein displaying the interactions with amino acid residues.

The molecular docking results for the individual compounds obtained from the extracts are presented in Table 5. The docking simulations displayed compound-amino acids interactions such as hydrogen bonding, Pi-Pi stacked, Van der Waals interactions (Figs. 4 and 5). O–H of compounds 1 and 5 formed hydrogen bonding with the amino acid residues PHE338 while other amino acid residues formed Van der Waals interactions (Figs. 4 and 5). Compound 5 showed the highest affinity for SARS-CoV-2 protein compared to other compounds with binding energy of − 6.8 kcal mol^−1^ this may be traced to its high stability in the binding pocket of the enzyme during binding compared to other compounds. Aside from compounds 1 and 2 some of the studied compounds docked to the active site but with a lower binding energy while some did not bind appropriately which may be due to the binding conformation of these compounds around the binding pockets. The molecular docking protocol was validated by re-docking with Baricitinib (reference drug) with selected protein into the same binding site of the protein (Fig. 6). The results showed similar binding energy with some of the compounds under study especially compound 5. Similar interaction patterns were also observed but interacted via Pi-Pi bonding with the amino acid residue PHE338.

**Table 5:** Calculated binding energies with the respective targets obtained from AutoDock Vina.

S/N	Compounds	kcal/mol
1		− 6.4
2		− 6.2
3		− 6.3
4		− 6.2
5		− 6.8
6		− 5.5
7		− 5.2
8		− 5.2
9		− 5.3
Baricitinib		− 6.5

## Discussion

This study showed that SARS-CoV-2 and other members of the coronavirus family are sensitive to PHELA. However, we also observed differences between these viruses’ susceptibilities to PHELA, with SARS-CoV-2 wild type as well as SARS-CoV showing higher sensitivity to the extract compared to the N501Y.V2 variant and MERS-CoV. Molecular docking analysis of the LC–MS identified different compounds in the extract that were shown in silico to bind the viral envelope spike. These data support the continued development of PHELA as a potential drug to treat COVID-19 and other coronavirus infections since this extract is likely to be effective against different members of this family of viruses.

PHELA showed inhibitory activity against the virus with IC_50_ in the low microgram range. It is noteworthy that even at the lowest concentration of PHELA tested there was still at least 30% inhibitory activity against the wild-type or Wuhan SARS-CoV-2. Furthermore, the extract was able to inhibit other strains of SARS-CoV-2 including the strain that caused the first wave of infections in South Africa, and the significantly mutated N501Y.V2 variant that was responsible for the second wave of infections in South Africa. In addition, the PHELA extract was as well active against other members of the coronavirus family, namely SARS-CoV that caused an epidemic in China in 2003^39^, and MERS-CoV that was responsible for an epidemic in the Middle East in 2017^40^. These data suggest that PHELA is likely to have a broad spectrum of activity against different SARS-CoV-2 viruses, and this is also likely to be true for other viruses of the coronavirus family. Note that our molecular docking data suggest that PHELA may inhibit the virus infection of cells via interaction with the envelope spike protein. The fact that MERS-CoV was many folds less sensitive to PHELA compared to SARS-CoV, as well as the different strains of SARS-CoV-2 tested, may be due to the differences in the envelop spike of these viruses. Furthermore, it is known that SARS-CoV and SARS-CoV-2 are genetically much closer to each other than they are to MERS-CoV^38,41^. The above observation is also supported by the fact the N501Y.V2 with its significant S protein mutations also showed a marked reduction in sensitivity to PHELA compared to the wild-type SARS-CoV-2 viruses.

It will be interesting to do a direct comparison of the PHELA extract activity against SARS-CoV-2 with other known entry inhibitors such as antibodies and lectins. This is also true for lectins such as Griffiths in that they have been tested against other coronaviruses closely related to SARS-CoV-2^42,43^. One way that PHELA’s activity can be improved is by its further purification. This is because in a crude or semi-purified extract it is possible that some elements may be obstructing the inhibition of the virus by the active compounds. The phytochemicals in PHELA may be synergistic and purification may concentrate these compounds as well and make the extracts more active. Such purification may also help eliminate the small toxicity observed with PHELA when used at high concentrations, as suggested by its TC_50_ value.

PHELA’s inhibitory activity against SARS-CoV-2 suggests that it has potential for further development for use against COVID-19. These data also suggest that the extract potentially contains compounds that have activity against other coronaviruses, such as MERS-CoV^41^. Note that some investigators have already shown the potential use of herbal medicine to treat COVID-19 and other viral infections such as the Japanese encephalitis virus and HIV-1^42–44^. This is particularly important given that currently most treatments against COVID-19 are based on alleviating symptoms, while except for antibodies from plasma of previously infected individuals^45^, there is no drug that directly clears the virus or prevents its entry into target cells.

## Conclusion

The study found that PHELA in vitro inhibited the growth of SARS-CoV-2 and SARS-CoV infection and MERS-CoV with the implication for further development of PHELA for the infection controls of these viruses. In silico docking studies of compounds identified in PHELA showed very strong binding energy interactions with the SARS-COV-2 proteins. Compounds 1 and 5 showed the highest binding affinities for SARS-CoV-2 protein compared to other compounds and its comparable to the reference drug (Baricitinib). These studies could help direct us to unravel the possible mechanism of action of PHELA against COVID-19 or the SARS-COV-2 infection. Our data showed that PHELA has the potential for further development as a COVID-19 therapeutic. This herbal product could be developed as a complementary treatment therapy for COVID-19. PHELA could be developed as a standalone or adjunctive therapy for COVID-19 with the standard of care. However, these require further experimental validation in SARS-Cov-2 infection models and COVID-19 patients as well as on the different variants of SARS-COV-2, especially those variants of concern.

## Data Availability

All data generated or analyzed during this study are included in this published article and available from the corresponding author on reasonable request.
